# Fluid Management in Patients with Concomitant Acute Brain Injury and ARDS

**DOI:** 10.3390/jcm15218675

**Published:** 2026-11-06

**Authors:** Esenia Bechir, Aristomenis Exadaktylos, Wolf Hautz, Mairi Ziaka

**Affiliations:** 1 Department of Emergency Medicine, Inselspital, University Hospital, University of Bern, 3010 Bern, Switzerland; 2 Academic Department of Emergency Medicine, School of Medicine, University of Cyprus, 1678 Nicosia, Cyprus; 3 Service des Urgences-SMUR, Fribourg Cantonal Hospital (HFR), 1708 Fribourg, Switzerland

**Keywords:** acute brain injury, acute respiratory distress syndrome, fluid management, fluid overload, fluid responsiveness, hypervolemia, hypovolemia, lung injury

## Abstract

The main goals of therapeutic management in patients with acute brain injury (ABI) are to maintain cerebral perfusion pressure (CPP), promote cerebral oxygenation, reduce cerebral edema, prevent intracranial hypertension, and minimize acute neuronal injury in order to limit secondary brain damage. In this context, fluid management is of vital importance, as hypotension and hypovolemia are common in patients with ABI, predisposing them to secondary injury and poorer outcomes, and often necessitating a more liberal fluid strategy. On the other hand, ABI frequently leads to lung injury (LI), including neurogenic pulmonary edema (NPE) and acute respiratory distress syndrome (ARDS), which typically require a conservative fluid strategy to limit pulmonary edema, thereby making fluid resuscitation a significant clinical challenge. Hypervolemia and fluid overload are associated with serious complications, including worsening cerebral edema, delayed ischemic deficits, hemorrhagic diathesis, congestive heart failure, and pulmonary edema. Evidence to guide fluid management strategies in patients with concomitant ABI and ARDS remains limited. Current guidelines recommend targeting normovolemia in patients with ABI using a multimodal approach, with primary endpoints such as arterial blood pressure and fluid balance, while avoiding a negative fluid balance. In these patients, an individualized fluid therapy based on hemodynamic and clinical parameters, as well as cerebral dynamics, is recommended, taking into account the disease course and the potential ARDS subphenotypes.

## 1. Introduction

The general term “acute brain injury” (ABI) refers to various etiologies of acute brain damage, including traumatic brain injury (TBI), acute ischemic stroke (AIS), subarachnoid hemorrhage (SAH), and intracranial bleeding, all of which are associated with significant morbidity and mortality, often due to extrapulmonary complications [1]. Recent studies have convincingly highlighted the existence of an invisible, bidirectional biological communication between the brain and peripheral organs, including the lungs, heart, kidneys, gastrointestinal tract, liver, and lymphatic system, aimed at maintaining homeostasis; however, this communication can also contribute to acute or chronic impairment of individual or multiple organs [1,2,3,4]. It is widely assumed that the lungs are the most susceptible to secondary injury in patients with ABI, with pneumonia, often related to aspiration and/or ventilator use, neurogenic pulmonary edema (NPE), and ARDS representing the most common respiratory complications [1,5,6]. Notably, it has been shown that ARDS complicates the clinical course of 38% of patients with non-traumatic SAH, 30% of those with TBI, 28% with spontaneous intracerebral hemorrhage, and 4% with AIS [7].

The main characteristics of ARDS include severe dyspnea, hypoxemia, impaired lung compliance, and diffuse pulmonary opacities, which cannot be explained by cardiogenic pulmonary edema. Since its initial description, the definition of ARDS has been revised multiple times to better characterize these features and more precisely assess the severity of hypoxemia [8,9,10,11]. Recently, given advances in ARDS treatment and ongoing research, a new global definition has been introduced. It includes the use of high-flow nasal oxygen (≥30 L/min) for hypoxemia support, incorporates pulse oximetry (ratio of peripheral oxygen saturation (SpO_2_) to inspired oxygen fraction (FiO_2_), SpO_2_: FiO_2_  ≤  315) for assessing hypoxemia severity, and recognizes lung ultrasound as an adequate tool for detecting bilateral opacities, especially when access to advanced imaging modalities is constrained [12].

ARDS is characterized by diffuse alveolar damage, alveolo-capillary hyperpermeability, and susceptibility to hydrostatic forces related to intravascular volume, leading to fluid accumulation in the alveolar spaces, severe respiratory failure, and reduced lung compliance. Accordingly, a cornerstone of ARDS management is the adoption of a conservative fluid strategy, guided by close monitoring, to limit alveolar fluid buildup and thereby reduce ventilator days, intensive care unit (ICU) and hospital stays, and mortality, as supported by growing evidence [13,14,15]. Otherwise, in patients with ABI, hypovolemia and systemic hypotension are major contributors to secondary brain injury, as they reduce CPP, promote cerebral vasodilation and intracranial hypertension, and increase the risk of ischemic injury, making fluid resuscitation a cornerstone of neurocritical care to maintain euvolemia and hemodynamic stability [16,17,18,19]. However, it should not be overlooked that fluid overload is associated with cerebral edema, increased intracranial pressure (ICP), and worsening neurologic outcomes [20]. To complicate matters further, when ABI and ARDS coexist, the complexity of care increases substantially, requiring a precise and often personalized approach to supportive therapies. Among these, fluid management plays a central role in critical care, as it directly affects both pulmonary and cerebral outcomes [7]. This presents a significant clinical dilemma: while a restrictive fluid strategy is beneficial for lung protection in ARDS, it may compromise CPP in patients with ABI. Conversely, liberal fluid resuscitation aimed at preserving cerebral perfusion can exacerbate pulmonary dysfunction in the context of ARDS. The coexistence of these conditions demands a thorough understanding of the underlying pathophysiology and a highly individualized fluid management approach, guided by continuous hemodynamic and multimodal monitoring, including transpulmonary thermodilution, bedside ultrasound, and ICP monitoring [21]. The selection of fluid type, the volume administered, and the patient’s response to diuretics or vasopressors are all critical factors that must be carefully considered in light of both respiratory and neurological outcomes [22]. However, despite the clinical importance of this intersection, there is a relative paucity of high-quality evidence and clear guidelines specifically addressing fluid management in patients suffering from both ARDS and ABI. Most studies and recommendations focus on each condition in isolation, creating a potential gap in optimal care when these pathologies coexist [23]. Moreover, fluid responsiveness and tolerance may vary significantly between individuals, necessitating dynamic and adaptable strategies rather than rigid protocols [15].

The purpose of the present work is to describe and analyze current literature, clinical evidence, and expert recommendations regarding fluid management in critically ill patients with ARDS and ABI. This work will explore the physiological interactions between the respiratory and central nervous system (CNS) in the context of fluid therapy and highlight areas of consensus and controversy in current practice. It aims to provide a framework for individualized fluid management that balances the competing demands of pulmonary and cerebral protection, thereby contributing to the development of safer, more effective treatment strategies for this highly vulnerable ICU population.

## 2. Methods

A comprehensive literature search was conducted in the PubMed database to identify relevant articles focusing on fluid management in critically ill patients with ARDS and concomitant ABI. The search was performed using specific keywords to capture studies related to our research topic. The keywords used included: “fluid management,” “acute brain injury,” “traumatic brain injury,” “subarachnoidal hemorrhage,” “intracerebral hemorrhage,” “acute ischemic stroke,” “intracranial pressure,” “ARDS,” “lung injury,” “neurocritical care,” “lung protective strategies,” “cerebral perfusion,” and “hemodynamic monitoring”. Boolean operators (AND, OR) and truncation were strategically employed to enhance and refine search results. The search focused on English-language publications from 2010 through 2026. Earlier seminal studies were also considered where relevant to establish the historical background and incorporate foundational evidence. The literature search was last updated on 15 October 2026.

The literature search covered peer-reviewed clinical and experimental studies, systematic reviews, clinical guidelines, and expert consensus statements. Eligible publications addressed adult patients with concomitant ABI, including TBI, SAH, or intracerebral hemorrhage (ICH), and ARDS. Studies involving pediatric populations, case reports, or publications in languages other than English were excluded. Study selection involved an initial assessment of titles and abstracts, followed by full-text evaluation of potentially relevant articles. In addition to original investigations, systematic reviews, meta-analyses, and narrative reviews were considered to contextualize the evidence, trace developments in the field, and identify influential contributions. Expert-authored narrative reviews further informed the interpretation of existing findings, particularly regarding unresolved controversies and gaps in knowledge. The available preclinical, translational, and clinical evidence was synthesized qualitatively and organized thematically, without applying a formal systematic-review methodology.

## 3. Pathogenesis of ARDS

ARDS is a heterogeneous syndrome characterized by an inflammatory response in the lungs due to an acute pathological insult. It is responsible for one in 10 admissions to ICUs and one in four mechanical ventilations [24]. Although the pathophysiology of ARDS is beyond the scope of the current review, it should be emphasized that inflammatory reactions play a significant role in its pathogenesis, leading to damage of the microvascular endothelium and alveolar epithelium of the alveolar-capillary barrier, which results in increased vascular permeability and subsequent edema [25]. The pathogenesis develops through three sequential phases that have overlapping characteristics: (1) the acute exudative/inflammatory phase, (2) the proliferative phase, and (3) the fibrotic phase [26,27]. During the acute phase, both endothelial and epithelial damage occur in the alveoli and capillaries, with alveolar macrophages releasing cytokines like interleukin-1, 6, 8, and 10 (IL-1, 6, 8, and 10), along with tumor necrosis factor alpha (TNF-a). Immunomodulatory proteins play a crucial role in activating neutrophils, which then produce pro-inflammatory substances. They also promote fibroblasts to generate the extracellular matrix. As a result, the permeability of the alveolar-capillary membrane increases, leading to the accumulation of protein-rich edema in both the alveoli and the interstitium. This disruption of pulmonary oncotic pressures makes the alveoli more vulnerable to higher hydrostatic pressure, potentially leading to the development of non-cardiogenic pulmonary edema [28].

In the proliferative phase, type II pneumocytes refill the alveoli; alveolar edema is alleviated through the active transport of sodium and chloride and the function of water channels, and protein is eliminated from the small airways, helping to restore the structure and function of the alveoli [28]. Some patients with ARDS may enter the fibrotic phase, which is marked by the gradual remodeling and resolution of granulation tissue in the alveoli and interstitial areas. This phase can occur unexpectedly and significantly delay recovery of lung function. Unfortunately, the presence of fibrosis is associated with increased mortality rates, making it critical to monitor and manage ARDS patients effectively to improve their outcomes [28,29].

## 4. Pathophysiology of ARDS in Patients with ABI

**Figure 1:**
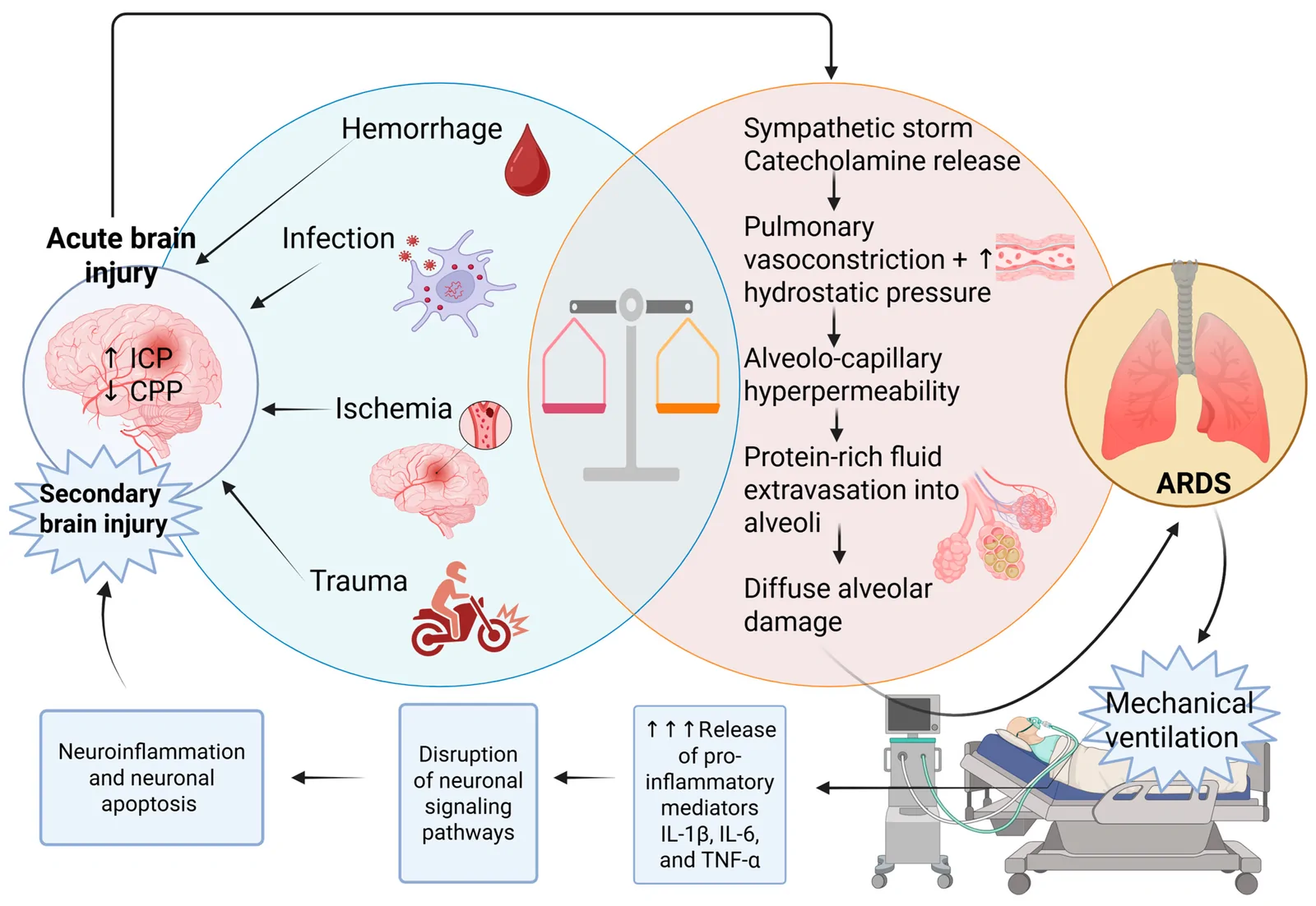
Schematic overview of the bidirectional pathophysiological interactions between ABI and ARDS. ABI resulting from trauma, ischemia, infection, or hemorrhage leads to increased ICP and reduced CPP. Sympathetic activation with excessive catecholamine release induces pulmonary vasoconstriction, increased hydrostatic pressure, and enhanced alveolo-capillary permeability, resulting in protein-rich fluid extravasation and diffuse alveolar damage. The development of ARDS and the need for mechanical ventilation further exacerbate systemic inflammatory responses, thereby contributing to the development of secondary brain injury. ABI: acute brain injury; ARDS: acute respiratory distress syndrome; CPP: cerebral perfusion pressure; IL: interleukin; ICP: intracranial pressure; TNF: tumor necrosis factor.

ABI, such as TBI, ICH, SAH, and AIS, is a significant cause of global morbidity and mortality [1]. It has been convincingly demonstrated that ABI is a systemic process that extends beyond the CNS, affecting extracranial organs and systems, including the lungs, heart, kidneys, lymphatic system, and liver [1,2,3,5,30,31]. Among these, the lungs are the most susceptible to the secondary effects of ABI, with pulmonary complications including NPE, ARDS, and pneumonia occurring frequently in neurocritically ill patients [1]. The largely invisible crosstalk between the brain and lungs in ABI is driven by complex, multifactorial pathophysiological mechanisms, including immune and inflammatory reactions, altered neurotransmission, and disturbances of the autonomic nervous system and catecholamine release (Figure 1) [32,33,34].

Among patients with ABI, ARDS has a high incidence, ranging from 20% to 30% [35]. The mechanisms of ABI-induced ARDS include sympathetic activation and a catecholamine surge in the systemic circulation, leading to rapid shifts of large volumes of blood from the systemic to the pulmonary circulation and a subsequent elevation of hydrostatic pressure, which contributes to fluid extravasation into the interstitial and alveolar spaces (Figure 1) [36,37,38]. Additionally, hemodynamic changes associated with a catecholamine storm and systemic arterial hypertension, often used therapeutically to preserve CPP, may further exacerbate pulmonary fluid accumulation [39,40]. Furthermore, the double-hit theory presented by Mascia et al. (2009) suggests that inflammatory cascades related to ABI create a systemic inflammatory milieu (‘first hit’), which predisposes peripheral organs and systems to injury from secondary processes associated with inflammation, such as infections and surgical procedures (‘second hit’) [6]. Finally, Ziaka and Exadaktylos (2024) have proposed the ‘triple-hit’ hypothesis, which, in addition to the ‘double-hit’ theory, incorporates dysbiosis and intestinal dysfunction associated with ABI (‘third hit’), leading to systemic inflammation, immunosuppression, and bacterial translocation, which are major contributors to LI and ARDS [3].

## 5. Pathophysiology of ABI

### Role of Intracranial Pressure, Cerebral Perfusion Pressure, and Brain Edema

The brain is enclosed within the rigid confines of the skull, which means its volume is fixed. Under normal circumstances, it receives approximately 15% to 25% of the cardiac output, with cerebral blood flow (CBF) ranging from 40 to 50 mL per 100 g of brain tissue per minute. CBF is directly proportional to CPP and inversely proportional to cerebrovascular resistance (CVR), as expressed by the equation CBF = CPP/CVR. Changes in arterial diameter due to vasoconstriction or vasodilation during autoregulation alter CVR and, consequently, CBF [41]. Additionally, CPP represents the net pressure gradient driving oxygen delivery to brain tissue and is calculated as the difference between mean arterial pressure (MAP) and ICP. When ICP rises excessively, CPP decreases, potentially leading to cerebral ischemia and neuronal injury [2]. In healthy subjects, compensatory mechanisms maintain CPP within a stable range (50–150 mm Hg) by increasing cerebrovascular resistance when MAP and CPP rise, and decreasing it when MAP and CPP fall [42]. However, this homeostatic compensation is frequently compromised in ABI, making the brain susceptible to systemic hemodynamic fluctuations [43,44,45].

Intracranial hypertension can be significantly worsened by the presence of fluid excess in the brain parenchyma [46]. Depending on its intracellular or extracellular location and the integrity of the blood–brain barrier (BBB), cerebral edema can be categorized as cytotoxic or vasogenic. Ionic edema represents an intermediate form, resulting from water and electrolyte shifts across an intact BBB and associated with decreased interstitial osmolality, thereby pathophysiologically linking it to the development of cytotoxic edema [47,48]. Cytotoxic edema refers to cellular swelling of astrocytes and neurons following the uptake of ions, mainly sodium, which drives water movement from the extracellular to the intracellular space, typically after events causing adenosine triphosphate (ATP) depletion and mitochondrial dysfunction [47,49]. Vasogenic edema results from the influx of water and albumin across a disrupted BBB due to abnormalities in tight junctions, leading to extracellular fluid accumulation [47,49]. The presence of edema further compresses brain structures and impairs the autoregulation of CBF, creating a vicious cycle that exacerbates brain injury. The mechanism of damage from this condition can be divided into two phases: (1) a primary acute injury resulting from the traumatic event, and (2) a secondary injury caused by hypotension and hypoxia that occur due to the initial injury, leading to ischemia and necrosis of neural cells [50].

## 6. Pathophysiological Crosstalk Between Hypervolemia, Acute Brain Injury, and Lung Damage

Fluid overload is commonly observed during critical illness and can affect multiple organs, with the lungs being particularly vulnerable, leading to oxygenation insufficiency, pulmonary edema, and ARDS [14,51,52,53]. Fluid overload may result in extravascular and interstitial pulmonary edema, partly due to increased hydrostatic pressure, resulting to altered gas exchange and impaired oxygenation [54,55]. Recent experimental research suggests that a positive fluid balance is associated with decreased end-expiratory lung gas volume and impaired oxygenation, which may be related to the development of ascites [56]. In addition, it has been convincingly demonstrated in recent years that, in the pathophysiology of interstitial edema, stimulated pro-inflammatory reactions in the pulmonary endothelium may contribute to elevated intravascular pressure and further aggravate interstitial edema [55,57,58].

Traditionally, the Starling model explains transvascular fluid exchange as a balance between hydrostatic and oncotic pressure gradients [59]; however, recent advances highlighting the role of the glycocalyx have called this theory into question [60]. Indeed, current research has led to a revised Starling equation that incorporates the roles of the endothelial glycocalyx layer, a network-like structure of glycosaminoglycans (GAGs), proteoglycans, and glycoproteins lining the alveolar surface, the endothelial basement membrane, and the extracellular matrix [59,61]. The GAGs in the glycocalyx are thought to form a charged network over cell–cell junctions, contributing to the transvascular oncotic pressure gradient and playing a role in the Starling mechanism that regulates fluid movement [62]. Vital functions of the glycocalyx include maintaining endothelial integrity and regulating processes such as coagulation, cell signaling, and inflammation [63]. Critical vascular functions, such as inhibiting leukocyte adhesion, mechanotransducing shear stress, and regulating vascular tone, are supported by the pulmonary endothelial glycocalyx [64].

Several studies have supported the “modified Starling” theory, which posits that the endothelial surface layer, rather than the cell–cell junctions, governs the transvascular oncotic gradient. These studies showed that degradation of the glycocalyx increased protein and fluid permeability [65,66]. Notably, evidence from clinical and experimental research suggests that hypervolemia and fluid overload may result in shedding and degradation of the endothelial glycocalyx, leading to increased vascular permeability and tissue edema [67,68,69], indicating a potential glycocalyx-guided fluid management strategy based on glycocalyx degradation products [68].

On the other hand, hypervolemia-associated worsening of oxygenation in patients with lung injury (LI) or ARDS may lead to further neurological deterioration and secondary brain injury through the complex crosstalk between the brain and lungs [15,70]. Indeed, hypoxia, along with hemodynamic alterations, is a well-characterized risk factor for the aggravation of existing brain injury and is associated with poor outcomes [71]. Furthermore, cognitive dysfunction was significantly associated with a mean PaO_2_ of 72 mmHg, compared with a mean PaO_2_ of 86 mmHg in mechanically ventilated survivors of LI [72]. The main regulators ensuring adequate brain oxygenation, that is, brain oxygen availability (DO_2_), are appropriate CBF and adequate arterial oxygen content (CaO_2_) [73] as DO_2_ is determined by the product of CBF and CaO_2_ [73,74]. Thus, to preserve brain oxygen availability, CBF increases through cerebral vasodilation as a compensatory mechanism in the presence of hypoxia [74]. However, brain tissue oxygenation is a dynamic and multifactorial process that depends on arterial oxygen tension, systemic oxygen delivery, oxygen transport from the capillary network to the mitochondria, subsequent mitochondrial oxygen metabolism, hemoglobin concentration, cardiac function, pH, and body temperature [74,75,76].

On the other hand, although not within the scope of the present review, it is well established that LI and ARDS are characterized by local and systemic inflammatory processes that may exert detrimental effects on extrapulmonary organs, including the brain [77]. Lung microvascular barrier dysfunction and endothelial and epithelial hyperpermeability, particularly in the presence of hypervolemia and fluid overload, further enhance the systemic inflammatory milieu [78,79]. Experimental research in animal models of LI highlights that lung-derived inflammatory mediators may induce neuroinflammatory responses and secondary brain injury; however, the existing data remain limited [80].

Mechanical ventilation, frequently applied in patients with LI and ARDS, can result in secondary brain injury either through the massive release of pro-inflammatory mediators, including IL-1β, IL-6, and TNF-α, or by altering vagal signaling with subsequent neuroinflammation and neuronal apoptosis [70]. The extent of brain injury appears to be dose-dependent, increasing with higher tidal volume, driving pressure, and mechanical power [70,81,82]. To complicate matters further, experimental research in anesthetized, mechanically ventilated rats with LI has demonstrated that hypervolemia leads to an increased lung wet-to-dry (W/D) ratio, accompanied by worsening oxygenation and an increase in static lung elastance, alveolar and endothelial cell damage, and elevated levels of pro-inflammatory mediators such as IL-6 [83], potentially further exacerbating primary brain injury. In addition to the hypervolemia-mediated inflammatory reactions observed in mechanically ventilated individuals, it can be hypothesized that the inflammatory responses associated with venous congestion, a typical feature of hypervolemic states, may play a pathogenetic role in worsening neuroinflammation. Indeed, experimental models of venous congestion have demonstrated a significant release of inflammatory mediators such as plasma IL-6, endothelin-1 (ET-1), and angiotensin II (AII) [84].

## 7. Hypotension in Acute Brain Injury

Hypotension frequently accompanies TBI, whereas in patients with SAH and AIS it is considered a rare complication [85,86]. Causative factors for the development of hypotension in patients with ABI include catecholamine storm and sympathetic overstimulation following the initial event, leading to systemic vasoconstriction or vasoplegia, as well as increases in cardiac afterload, myocardial workload, and oxygen demand. Catecholamine storm may further result in neurogenic heart cardiomyopathy and even cardiogenic shock through brain–heart crosstalk, involving brain areas such as the brainstem, insula, cortex, and hippocampus [2,85,86,87,88,89]. Moreover, particularly in patients with TBI, hemorrhagic shock due to extracranial blood loss is the leading cause of hemodynamic compromise and significant hypovolemia, resulting in neurological deterioration. Nevertheless, it is important to note that severe hypovolemia in neurocritical care patients may still be masked by normotensive arterial blood pressure due to the activation of autonomic compensatory mechanisms [85]. Indeed, immediately following a bleeding event, there is an excessive catecholamine release, including epinephrine and norepinephrine, leading to a 10- to 40-fold elevation of their concentrations. This is followed by compensatory activation of the renin-angiotensin system, resulting in vasoconstriction to preserve normotension [90,91]. At the initial stages of hemorrhagic shock, clinical signs include increases in heart and respiratory rate and decreases in pulse pressure, followed later by reductions in systolic arterial pressure and MAP. Compensatory tachycardia helps maintain cardiac output and MAP, even in cases of excessive blood loss (>30%) during the early stages of shock, despite reductions in stroke volume and venous return [92]. In cases of uncontrolled hemorrhage with large blood losses, the initial sympathomimetic phase is abruptly followed by the sympathoinhibitory phase (phase II). This phase is characterized by loss of vasoconstriction, enhanced catecholamine release, vasodilation with decreased vascular resistance, severe hypotension, and relative or absolute bradycardia, resulting in worsening shock and significantly impaired organ perfusion [91,92,93]. Hypotension, particularly when arterial blood pressure falls below 90 mmHg, has devastating effects in patients with TBI, leading to increased mortality and long-term morbidity [85,94,95].

## 8. Individualized Fluid Management in Acute Brain Injury and ARDS

In patients with ABI, including TBI, SAH, ICH, and AIS, cerebral autoregulation is often impaired, resulting in compromised brain oxygenation. Fluctuations in systemic hemodynamics, both hypotension and hypertension, can markedly affect CBF, making these patients particularly vulnerable to secondary brain injury [48,96,97]. Systolic blood pressure targets in ABI vary according to the underlying diagnosis and should be individualized based on patient physiology [48,96].

Individualization of volume therapy in clinical practice marks a significant shift from standardized fluid protocols to a dynamic, patient—tailored approach. This concept supports fluid administration based on continuous physiological assessment, the phase of disease [98,99] and patient-specific factors including chronic comorbidities (end-stage kidney disease, heart failure, right ventricular failure), body habitus (adjusted or ideal body weight may be used in obese patients), and resource setting (limited respiratory support availability increases risks of fluid overload) [100,101]. Moreover, only 50–57% of critically ill patients are fluid responsive at presentation, meaning nearly half will not benefit and may be harmed by additional fluid administration [102].

Fluid management in critically ill patients progresses generally through four distinct phases: an initial resuscitation phase involving rapid fluid administration, followed by an optimization phase focused on achieving adequate organ and tissue perfusion, then a stabilization phase maintaining physiologic homeostasis, and finally an evacuation phase or *de-escalation* during organ recovery, which typically requires active fluid removal [99,101,103]. The European Society of Intensive Care Medicine (ESICM) 2025 clinical practice guideline on fluid therapy for adult critically ill patients recommends administering up to 30 mL/kg of intravenous crystalloids during the initial phase of resuscitation. It further emphasizes the importance of making adjustments based on the clinical context and conducting frequent reassessments [104]. During the optimization phase, an individualized approach should be taken. However, the experts could not provide a recommendation for or against either restrictive or liberal fluid strategies during this phase [100].

During de-escalation, fluid removal must be adapted to the patient’s hemodynamic status, as excessive reduction in circulating volume can impair cardiac output and blood pressure [105]. However, the use of diuretics to manage hypervolemic states in patients with ABI should be approached with caution, given the extremely limited data on their use in this vulnerable patient population. Moreover, both insufficient [106] and excessive rates of fluid removal have been associated with increased mortality. Overall, these findings highlight the importance of a patient-specific approach to fluid management and the need for better predictors of tolerance to fluid removal [107].

In patients with concomitant ABI and ARDS, individualization is crucial due to competing therapeutic priorities: under-resuscitation threatens cerebral perfusion, while over-resuscitation can worsen pulmonary edema and may increase ICP [99,108,109]. The target should be normovolemia guided by multimodal monitoring of both lung physiology (compliance, oxygenation) and cerebral parameters (ICP, CPP).

All unstable patients, as well as those at risk and hospitalized in the ICU, should receive at least electrocardiography and invasive arterial blood pressure monitoring. Additionally, echocardiography and advanced invasive hemodynamic monitoring such as transpulmonary thermodilution to assess cardiac output, preload, afterload, and global systemic perfusion can guide hemodynamic management while considering CBF, ICP, CPP, and oxygenation. These parameters should be interpreted in relation to the clinical diagnosis and disease stage [48,110]. Fluid responsiveness refers to the left ventricle’s ability to enhance stroke volume following fluid administration [48,111]. The 2026 Surviving Sepsis Campaign guidelines suggest using dynamic measures to guide fluid resuscitation over physical examination or static measures alone [100].

Dynamic parameters outperform static indices in predicting fluid responsiveness. Pulse pressure variation (PPV > 10–12%) and stroke volume variation (SVV > 12%) show good diagnostic accuracy [101,102], although PPV thresholds may be lower in ARDS depending on PEEP [112], lower tidal volumes [113], spontaneous breathing or arrhythmias [114], whereas adjustment for pleural pressure further improves performance [115]. Nevertheless, a recent systematic review and meta-analysis of five studies involving 474 critically ill patients found that changes in absolute PPV accurately predicted fluid responsiveness during passive leg raising (PLR) in sedated, mechanically ventilated patients receiving low tidal volumes and without cardiac arrhythmias [113]. PLR increases venous return and represents an effective method for predicting fluid responsiveness [116,117]. Nevertheless, its use requires real-time cardiac index measurement [118] and raises concerns in patients with severe brain injury because of the potential risk of increased ICP and impaired cerebrovascular reactivity associated with increases in cerebral blood volume [116,117]. Indeed, a recent prospective observational study in patients with stable ABI reported disturbances in cerebrovascular autoregulation lasting up to 10 min after PLR, despite stable ICP, drawing our attention to the safety of this maneuver in patients with ABI [119]. Furthermore, point-of-care ultrasound (POCUS) facilitates bedside evaluation of cardiac function, stroke volume, and fluid responsiveness, with randomized evidence indicating improved outcomes when incorporated into resuscitation strategies [120,121,122].

Transcranial Doppler (TCD) represents the most commonly applied non-invasive technique, as it measures CBF velocities in the middle cerebral arteries and simultaneously enables evaluation of autoregulation through the mean velocity index (Mx). This parameter is calculated from the correlation between flow velocity and MAP [123,124]. Moreover, near-infrared spectroscopy (NIRS) allows continuous monitoring of regional cerebral oxygenation, with derived oximetry indices [48,125]. Furthermore, brain tissue oxygen tension (PbtO_2_) monitoring provides a direct assessment of tissue oxygenation and, when PaO_2_ is kept stable, may serve as a surrogate marker of cerebral perfusion and metabolic balance [124]. At a broader integrative level, positron emission tomography using ^15^O tracers remains the reference standard for quantitative evaluation of CBF and oxygen metabolism, and studies in TBI have demonstrated substantial spatial heterogeneity and regional disturbances in flow–metabolism coupling [126,127].

Taken together, an integrated multimodal strategy combining neurological (ICP, CPP, PbtO_2_, TCD/NIRS), respiratory (lung mechanics, oxygenation, PaCO_2_), and hemodynamic (cardiac output, dynamic preload assessment, ultrasound-based volume evaluation) monitoring is recommended to maintain normovolemia while simultaneously optimizing cerebral perfusion and pulmonary function in ABI patients, including those with concomitant ARDS [30].

Metabolic markers such as changes in central venous-to-arterial PCO_2_ difference (ΔP(v–a) CO_2_) and in central venous oxygen saturation (ΔScvO_2_) may help identify fluid responsiveness when cardiac output monitoring is unavailable [128]. Point-of-care ultrasound complements assessment, with Inferior vena cava collapsibility (IVC)/Superior vena cava collapsibility (SVC) variability indicating responsiveness and pulmonary B-lines suggesting volume overload [102,129]. In contrast, static parameters such as central venous pressure have limited predictive value and should not be used in isolation.

## 9. Current Strategies in Fluid Management

### 9.1. Conservative vs. Liberal Fluid Therapy in Patients with ARDS

The most suitable fluid management strategy in patients with ARDS is unclear and remains debated. Although observational studies had already suggested more than 35 years ago that a liberal fluid strategy could be harmful in patients with ARDS [130], the seminal trial that defined fluid management in ARDS was the ARDS Network Fluid and Catheters Treatment Trial (FACTT), published in 2006 [14]. The study enrolled 1000 non-shock patients with ARDS to receive either a conservative or a liberal fluid strategy, guided by hemodynamic and clinical parameters such as central venous pressure (CVP) and pulmonary artery occlusion pressure (PAOP). The protocol included a stringent approach to active diuresis, fluid boluses, vasopressors, and/or inotropes. Although 60-day mortality was similar between groups, the study demonstrated that patients in the conservative treatment group had significantly lower daily cumulative fluid balance, more ventilator-free days, and more ICU-free days, without an increase in organ failure or need for renal replacement therapy, highlighting the safety of the conservative strategy [14]. These findings are in line with the results of a retrospective analysis from the Multi-Parameter Intelligent Monitoring in Intensive Care (MIMIC-II) project, underscoring positive fluid balance as a major risk factor for the development of ARDS [131]. In addition, a post hoc analysis of 244 surgical patients enrolled in the FACTT confirmed the findings of the original trial, demonstrating no significant differences in 60-day mortality; however, it highlighted more ventilator-free and ICU-free days in the conservative treatment group, with no differences in the incidence of renal failure [132]. Nevertheless, a further retrospective analysis of the FACTT showed that conservative fluid management reduces mortality in patients with low initial CVP, in whom intravenous fluid administration was associated with increased mortality [133]. This is consistent with the observation that distinct ARDS subphenotypes exhibit different responses to fluid management strategies, with the hyperinflammatory phenotype showing higher mortality if managed conservatively, whereas the hypoinflammatory subphenotype is likely to benefit from a conservative fluid strategy [27]. However, two small randomized control trials [134,135] failed to demonstrate improvements in mortality or ICU length of stay, although interpretation of their results is limited by small sample sizes and a serious risk of bias [136].

Based on the aforementioned findings, the Faculty of Intensive Care Medicine and Intensive Care Society Guideline Development Group (2019) provided a weak recommendation for a conservative fluid management strategy in patients with ARDS, due to the low quality of existing evidence and the fact that the guidelines are based on a single trial [136].

### 9.2. Fluid Management Strategies in Patients with Concomitant ABI and ARDS

**Figure 2:**
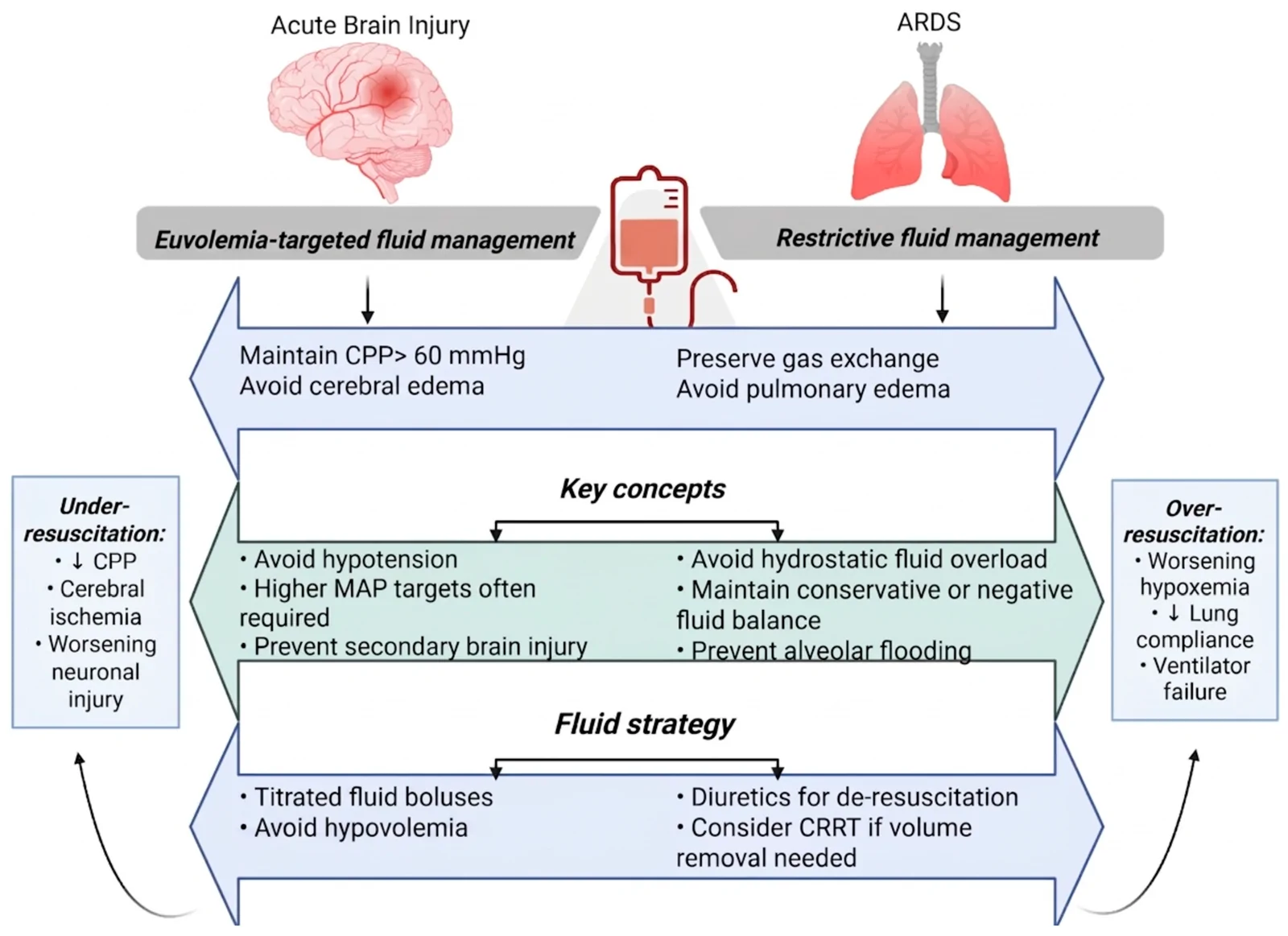
Conceptual framework for fluid management in patients with concomitant ABI and ARDS. While ABI favors a liberal fluid approach to maintain cerebral perfusion and prevent secondary brain injury, ARDS requires a restrictive strategy to preserve gas exchange and avoid pulmonary edema. An individualized, titrated fluid strategy balancing cerebral and pulmonary goals is essential to prevent both under- and over-resuscitation. ABI: Acute brain injury; ARDS: Acute respiratory distress syndrome; CPP: Cerebral perfusion pressure; CRRT: Continuous renal replacement therapy; MPP: Mean perfusion pressure.

In patients with concomitant ABI and ARDS, guiding a restrictive fluid management strategy while maintaining adequate cerebral perfusion represents a complex challenge, particularly in those with altered CBF or vasospasm (Figure 2) [7]. Indeed, a conservative fluid management strategy frequently leads to a decrease in circulating blood volume, which may reduce venous return and lower cardiac preload, and in cases of severe hypovolemia can result in arterial hypotension and inadequate organ perfusion [101,137,138]. Altered renal perfusion and acute kidney injury, anastomotic leakage, splanchnic ischemia, confusion, and multi-organ failure represent potential complications associated with hypovolemia. Importantly, impaired cardiac function may further reduce the patient’s ability to tolerate fluids, leading to elevations in cardiac filling pressures [98,137,139]. This is of crucial importance in patients with ABI, as the catecholamine surge observed in this population may lead to ventricular dysfunction, vasoplegia, and further hemodynamic instability and shock [140]. Moreover, a conservative fluid strategy has been associated with long-term cognitive impairment in patients with ARDS, as shown in a post-hoc analysis of survivors from the FACTT trial [72], a finding that is particularly important in neurocritical patients. Moreover, in patients with non-penetrating brain injuries and Glasgow Coma Scale scores of 3–8 after resuscitation, a fluid balance below −594 mL has been associated with poorer prognosis, independent of cerebral and systemic hemodynamic parameters, including MAP, ICP, and CPP [141].

The devastating effects of hypervolemia in patients with ABI are well recognized. In patients with aneurysmal SAH, hypervolemic states are associated with delayed ischemic deficits, vasospasm-related infarction, aggravation of cerebral edema, hemorrhagic infarction, longer hospital length of stay, and worse outcomes [142,143,144,145]. Moreover, the historic HHH therapy (hypervolemia, hemodilution, and hypertension) in patients with delayed cerebral ischemia following SAH leads to modest increases in systolic blood pressure, whereas notable decreases in hematocrit, with subsequent rheological consequences, are observed [146,147]. Indeed, Egge and co-authors (2001) reported that in patients with SAH randomized to receive either hypervolemic hemodilution or targeted normovolemia, those in the HHH group exhibited no benefits but experienced more complications, including hemorrhagic susceptibility, extradural hematoma, congestive heart failure, and arrhythmias [148]. Similarly, in patients with AIS who underwent endovascular thrombectomy, a higher positive fluid balance was associated with statistically significant poorer functional outcomes during the first 5 days and at discharge [149]. In a similar manner, in patients with TBI, hypervolemia may exacerbate cerebral edema, particularly in those with BBB disruption, and increase the risk of heart failure, pulmonary edema, and cardiogenic shock, while being associated with worse outcomes, including higher ICU mortality and poorer functional recovery [150,151].

Considering the complexity of managing patients with ARDS and ABI, and the detrimental effects of both hypovolemia and hypervolemia, the 2018 ESICM consensus statement on fluid therapy in neurointensive care [152] recommends maintaining normovolemia using a multimodal approach, with primary endpoints including arterial blood pressure and fluid balance, while avoiding a negative fluid balance. Additional parameters such as cardiac output, ScvO_2_, blood lactate, and urine output may further enhance the safety and optimization of fluid therapy. However, it should be noted that both the recommendation to target normovolemia and the recommendation against adopting a conservative fluid strategy and targeting a negative fluid balance were weak recommendations, reflecting the lack of high-quality evidence [152]. Nevertheless, the FACTT trial excluded patients with severe neuromuscular disease, defined as “disease that impairs ability to ventilate spontaneously, such as C5 or higher spinal cord injury, amyotrophic lateral sclerosis, Guillain-Barré syndrome, and myasthenia gravis” [14]. Consequently, it remains uncertain whether patients with severe ABI were included in the FACTT trial. Early use of vasopressors to increase MAP and optimize CPP, thereby supporting CBF and cerebral oxygenation, may be considered during the initial resuscitation phase, particularly in patients requiring large fluid volumes to achieve normovolemia [48,153,154,155,156].

In patients with ABI, cerebral perfusion targets are primarily derived from the Brain Trauma Foundation Guidelines by Carney et al. [16], which recommend treating intracranial ICP when it exceeds approximately 22 mmHg (range 20–25 mmHg) based on a synthesis of observational evidence and expert consensus. The same guideline, together with the randomized trial by Robertson et al. (1999) [157] supports maintaining CPP between 60 and 70 mmHg, as higher CPP targets (>70 mmHg) were associated with increased complications and no outcome benefit. In this seminal study, Robertson et al. included 189 adults admitted in coma following severe head injury and compared a CBF-targeted protocol, aiming to maintain CPP above 70 mmHg, with an ICP-targeted protocol, aiming to maintain CPP above 50 mmHg. Functional outcomes did not differ significantly between the groups at 3 months; however, ARDS developed in 15% of patients managed according to the CBF-targeted protocol, compared with 3.3% of those managed according to the ICP-targeted protocol, suggesting potential respiratory harm without demonstrable neurological benefit [157]. Consistent with these concerns, a study involving 18 patients with severe head injury found that epinephrine and dopamine administration were significantly associated with the development of ARDS, further suggesting a potential association between induced hypertension and pulmonary complications [158].

Multimodal neuromonitoring is supported by the BOOST II randomized controlled trial [159], which demonstrated that combining ICP and PbtO_2_ monitoring reduces the burden of cerebral hypoxia. Early observational studies on jugular venous oxygen saturation by Robertson et al. (1989) [160] identified values below 50% as indicators of cerebral ischemia. TCD studies by Czosnyka et al. [44] demonstrated that reduced diastolic flow velocity correlates with impaired cerebral perfusion.

Systemic physiological targets are based on evidence from the ARDS Network ARMA trial [161], which established lung-protective ventilation and oxygenation strategies, and from subsequent neurocritical care syntheses such as Robba et al. (2020) [30], which highlight the need to balance ARDS-directed and brain-directed goals. These studies support maintaining SpO_2_ >94% and PaO_2_ in a normoxic range of 80–140 mmHg in ABI patients, particularly when ARDS coexists. PaCO_2_ targets of 35–40 mmHg are recommended based on guideline consensus [16,30] with hyperventilation reserved for acute intracranial hypertension and permissive hypercapnia requiring close ICP monitoring. However, these findings should be interpreted in light of the fact that the ARMA trial excluded patients with intracranial hypertension and, therefore, may not be directly applicable to patients with ABI.

The neurocritical care guideline [16] recommends maintaining a systolic blood pressure of ≥100–110 mmHg, depending on age, to ensure adequate cerebral perfusion. In patients with concomitant ARDS, evidence indicates that moderate PEEP levels (up to ~12 cmH_2_O) are generally safe [30,162], but require careful monitoring of ICP, MAP, and CPP interactions. Clinical trial evidence addresses additional supportive physiological targets: normothermia, controlled glycemia, hemoglobin levels of 7–10 g/dL, and sodium balance [163,164,165,166,167,168,169].

## 10. Types of Fluids

Intravenous fluid administration is one of the most frequently performed interventions in the ICU. Fluids can be administered as electrolyte solutions, such as crystalloids, which distribute between the intravascular and interstitial compartments, or as colloid solutions including albumin containing large molecules that, under normal physiological conditions, tend to remain within the intravascular compartment for longer periods [170,171]. Consequently, colloids can produce greater intravascular volume expansion per unit of infused fluid and increase colloid osmotic pressure, potentially resulting in a volume-sparing effect [98,172]. However, the Saline versus Albumin Fluid Evaluation (SAFE) study, which included a heterogeneous population of critically ill patients, compared 4% albumin with saline and found no significant difference in overall mortality between the two groups [15,173]. Given the detrimental effects of hypervolemia across ICU populations, the potential volume-sparing effect of colloids may be especially relevant in patients with LI/ARDS [174,175] and ABI [150].

Evidence regarding the effects of albumin versus crystalloids in patients with ABI remains controversial, with some studies reporting improvements in cerebral and hemodynamic parameters [176,177], whereas others have reported adverse outcomes, including increased mortality [178]. The SAFE-TBI study, a post hoc analysis of the SAFE trial involving 460 patients with TBI, compared 4% albumin with saline for fluid resuscitation and found that albumin administration was associated with increased mortality. However, among patients with a Glasgow Coma Scale score of 9–12, mortality was higher in the saline group (21.6%) than in the albumin group (16%), although these subgroup findings should be interpreted cautiously [178]. In addition, a mechanistic post hoc analysis of the SAFE-TBI trial found that mortality was higher among patients receiving albumin than among those receiving saline when ICP monitoring was discontinued during the first week, but not when monitoring continued into the second week. The authors concluded that severe elevations in ICP were the most plausible mechanism underlying the increased mortality observed during the first week [179]. It is hypothesized that the tonicity of the solution is the determining factor associated with increases in ICP, rather than the use of albumin itself. Indeed, clinical studies in patients with SAH and AIS have reported improved neurological outcomes with high-dose albumin compared with crystalloids, suggesting potential neuroprotective properties [180,181,182]. These findings are further supported by experimental studies demonstrating that hypotonic albumin significantly increases ICP, without concomitant changes in left ventricular function or stroke volume, compared with isotonic albumin or normal saline [183].

Patients with ARDS frequently exhibit hypoproteinemia and consequently reduced serum albumin levels, which contribute to decreased plasma oncotic pressure and may predispose to pulmonary edema. Therefore, increasing intravascular oncotic pressure through albumin administration may theoretically limit interstitial edema formation, depending on the severity of endothelial barrier dysfunction [184,185]. However, data on albumin use in patients with ARDS remain limited. Martin et al. (2002) [186] conducted a prospective, randomized, double-blind study involving 37 hypoproteinemic, mechanically ventilated patients with LI. The study compared combined albumin and furosemide administration with placebo and found that albumin/furosemide therapy improved diuresis, fluid balance, oxygenation, and hemodynamic parameters [186]. A subsequent randomized, multicenter trial by the same group compared combined albumin and furosemide administration with placebo plus furosemide in 40 hemodynamically stable, hypoproteinemic, mechanically ventilated patients with LI/ARDS. Patients receiving albumin showed improved oxygenation, a greater negative fluid balance, and improved hemodynamic parameters [187].

Given that solution tonicity may influence the development of cerebral edema and increases in ICP, with potential implications for patient outcomes, the 2018 ESICM consensus recommends isotonic saline as the first-line fluid for neurocritically ill patients with hypotension [152]. Isotonic saline has a slightly higher osmolarity than plasma, whereas balanced crystalloids exhibit variable osmolarity depending on their composition, with Ringer’s lactate and Ringer’s acetate being slightly hypotonic. Importantly, balanced crystalloids have been associated with higher mortality in patients with TBI compared with normal saline [188,189], whereas evidence regarding their use in other causes of ABI remains scarce and is the subject of ongoing research [190].

Hypertonic saline has been investigated in various clinical settings involving shock, using concentrations ranging primarily from 3% to 20%, owing to its ability to induce rapid intravascular volume expansion with relatively small administered volumes [191]. This property may be particularly relevant in patients with ABI, in whom hypervolemia and positive fluid balance have been independently associated with increased mortality and poorer neurological outcomes [150,192]. In addition to its use for hemodynamic resuscitation, hypertonic saline is used in neurocritically ill patients to manage intracranial hypertension and correct hyponatremia associated with ABI [193,194]. A meta-analysis of five studies involving 175 patients with SAH showed that hypertonic saline at concentrations ranging from 3% to 23.5% reduced ICP by a mean of 8.9 mmHg. One of the included studies also suggested a potential improvement in clinical outcomes among patients with poor-grade aneurysmal SAH [195]. Beyond its effects on ICP and the development of cerebral edema and intracranial hypertension, hypertonic saline may contribute to improved functional outcomes by increasing cardiac output and cerebral perfusion, mitigating cerebral vasospasm, and alleviating glutamate-induced neurotoxicity and associated neuronal death [196]. However, studies investigating the administration of hypertonic saline to patients with TBI, either as a prehospital bolus or as a continuous infusion in the ICU, have failed to demonstrate improvements in short- or long-term outcomes [197,198].

Compared with mannitol for the management of elevated ICP, hypertonic saline may offer several advantages, including a faster onset of action, a greater and more sustained reduction in ICP, and potential efficacy in cases where mannitol has been ineffective [199]. Furthermore, hypertonic saline may avoid certain adverse effects associated with mannitol, such as hypovolemia and renal dysfunction [199]. Nonetheless, the available evidence on hypertonic saline is limited by differences in study design, methodological variability, small sample sizes, and heterogeneous patient populations. Accordingly, the 2020 guidelines from the Neurocritical Care Society suggest using hypertonic sodium solutions over mannitol for the management of intracranial hypertension or cerebral edema in patients with TBI, AIS, and ICH (conditional recommendation) [200]. During hyperosmolar therapy, close monitoring of serum sodium and serum osmolality every 4–6 h is explicitly recommended, with target values of less than 155 mEq/L for serum sodium and less than 320 mOsm/kg for serum osmolality, respectively [201].

## 11. Area of Future Research

As mentioned above, hypotonic 4% albumin solution may contribute to cerebral edema and intracranial hypertension, potentially worsening mortality and functional outcomes in patients with TBI, possibly through tonicity-associated translocation of albumin from the intravascular space into the brain interstitium due to altered BBB permeability [178,202]. High-concentration albumin solutions (e.g., 20–25%), with an osmolarity of ≥300 mOsm/kg, are used for plasma volume expansion in various conditions, including advanced liver disease and sepsis/septic shock [203]. The Lund concept advocates the slow administration of high-concentration albumin in patients with severe TBI, aiming to maintain euvolaemia and normal plasma oncotic pressure, with reported beneficial effects on ICP, cerebral perfusion, and local oxygenation [204,205]. Given the uncertainty of the current evidence, further well-designed randomized controlled trials are needed to evaluate the efficacy and safety of albumin administration, as well as procedure-related complications, including AKI. These trials should also address methodological heterogeneity, small sample sizes, and variability in functional outcome measures [140,205]. Further research should elucidate the pathophysiological mechanisms underlying the effects of albumin administration to improve patient selection and treatment outcomes. In addition, future trials investigating hyperoncotic albumin in patients with concomitant ABI and ARDS should incorporate close hemodynamic monitoring, neuromonitoring, and assessment of lung physiology, for example, using electrical impedance tomography (EIT).

No adequately powered prospective trial has investigated the effects of crystalloids in patients with non-traumatic SAH, particularly in the context of physiological vulnerabilities such as cerebral vasospasm. Further research is needed to evaluate the effects of crystalloids on vasospasm, morbidity, and mortality in this population. The ongoing CRYSTALLBrain trial, a multicentre, randomized, double-blind study, aims to compare normal saline with Ringer’s lactate in terms of the incidence of clinically relevant vasospasm in patients with SAH [190]. Its research framework could potentially be extended to patients with concomitant ABI and ARDS.

PLR is a simple bedside procedure for assessing fluid responsiveness. It induces an autotransfusion of approximately 300 mL of blood from the lower extremities towards the right heart, mimicking a rapid and reversible fluid challenge [206,207]. The potential of PLR as a useful method for assessing fluid responsiveness is well established, with recent data demonstrating that it increases inspiratory effort and thoracic blood volume while reducing end-expiratory lung impedance (EELI) [208]. However, its use in patients with ABI raises safety concerns regarding potential disturbances in cerebral autoregulation, cerebral edema, and intracranial hypertension [119]. Given the scarcity of existing evidence and the complexity of managing patients with coexisting ABI and ARDS [48], further well-structured studies are warranted to investigate the value of PLR in assessing fluid responsiveness in these patients under strict hemodynamic and neuromonitoring, thereby supporting an individualized, patient-oriented approach. Existing technologies such as quantitative electroencephalography (qEEG) could be evaluated, either alone or in combination with diagnostic modalities with established accuracy, such as TCD. Indeed, qEEG studies have demonstrated high diagnostic accuracy for detecting cerebral ischemia and may precede clinical deterioration by a median of 1–2.3 days [209,210]. Future studies could investigate whether integrating these monitoring modalities into fluid management strategies enables earlier detection of cerebral dysfunction associated with altered perfusion or volume status, before irreversible brain injury develops. Finally, recent preclinical research suggests that auditory stimulation, slow-wave activity, and glymphatic clearance may represent promising avenues for investigation in brain injury and provide a rationale for well-designed experimental studies [211,212].

## 12. Conclusions

Primary goals in the management of patients with ABI include maintaining adequate CPP to prevent secondary brain injury, which is often supported through intravenous fluid administration. However, the clinical course of patients with ABI is frequently complicated by the development of LI/ARDS, typically necessitating a conservative fluid management strategy to prevent further exacerbation of pulmonary edema. A standardized fluid resuscitation strategy for patients with concurrent ARDS and ABI does not exist. Current guidelines recommend targeting normotensive blood pressure and maintaining euvolemia, as both hypotension and hypo- or hypervolemia can have devastating consequences. Multimodal monitoring, including assessment of hemodynamic and cerebral parameters, is strongly recommended to guide therapeutic decisions and to avoid a negative fluid balance.

## Data Availability

The original contributions presented in this study are included in the article. Further inquiries can be directed to the corresponding author.
